# Risk of Perinatal and Maternal Morbidity and Mortality Among Pregnant Women With Epilepsy

**DOI:** 10.1001/jamaneurol.2024.2375

**Published:** 2024-08-05

**Authors:** Neda Razaz, Jannicke Igland, Marte-Helene Bjørk, K. S. Joseph, Julie Werenberg Dreier, Nils Erik Gilhus, Mika Gissler, Maarit K. Leinonen, Helga Zoega, Silje Alvestad, Jakob Christensen, Torbjörn Tomson

**Affiliations:** 1 Clinical Epidemiology Unit, Department of Medicine Solna, Karolinska University Hospital, Karolinska Institutet, Stockholm, Sweden; 2 Department of Global Public Health and Primary Care, University of Bergen, Bergen, Norway; 3 Department of Health and Caring Sciences, Western Norway University of Applied Sciences, Bergen, Norway; 4 Department of Clinical Medicine, University of Bergen, Bergen, Norway; 5 Department of Neurology, Haukeland University Hospital, Bergen, Norway; 6 Department of Obstetrics and Gynaecology, University of British Columbia and the Children’s and Women’s Hospital and Health Centre of British Columbia, Vancouver, British Columbia, Canada; 7 School of Population and Public Health, University of British Columbia, Vancouver, British Columbia, Canada; 8 National Centre for Register-Based Research, School of Business and Social Sciences, Aarhus University, Aarhus, Denmark; 9 Centre for Integrated Register-based Research, CIRRAU, Aarhus University, Aarhus, Denmark; 10 Knowledge Brokers, Finnish Institute for Health and Welfare, Helsinki, Finland; 11 Region Stockholm, Academic Primary Health Care Centre, Stockholm, Sweden; Karolinska Institute, Department of Molecular Medicine and Surgery, Stockholm, Sweden; 12 Centre of Public Health Sciences, Faculty of Medicine, University of Iceland, Reykjavik, Iceland; 13 School of Population Health, Faculty of Medicine and Health, UNSW Sydney, Sydney, Australia; 14 National Center for Epilepsy, Member of the ERN EpiCARE, Oslo University Hospital, Oslo, Norway; 15 Department of Neurology, Aarhus University Hospital, Aarhus, Denmark; 16 Department of Clinical Medicine, Affiliated member of EpiCARE, Aarhus University, Aarhus, Denmark; 17 Department of Clinical Neuroscience, Karolinska Institutet, and Department of Neurology, Karolinska University Hospital, Stockholm, Sweden

## Abstract

**Importance:**

Maternal epilepsy is associated with adverse pregnancy and neonatal outcomes. A better understanding of this condition and the associated risk of mortality and morbidity at the time of delivery could help reduce adverse outcomes.

**Objective:**

To determine the risk of severe maternal and perinatal morbidity and mortality among women with epilepsy.

**Design, Setting, Participants:**

This prospective population-based register study in Denmark, Finland, Iceland, Norway, and Sweden took place between January 1, 1996, and December 31, 2017. Data analysis was performed from August 2022 to November 2023. Participants included all singleton births at 22 weeks’ gestation or longer. Births with missing or invalid information on birth weight or gestational length were excluded. The study team identified 4 511 267 deliveries, of which 4 475 984 were to women without epilepsy and 35 283 to mothers with epilepsy.

**Exposure:**

Maternal epilepsy diagnosis recorded before childbirth. Prenatal exposure to antiseizure medication (ASM), defined as any maternal prescription fills from conception to childbirth, was also examined.

**Main outcomes and measures:**

Composite severe maternal morbidity and mortality occurring in pregnancy or within 42 days postpartum and composite severe neonatal morbidity (eg, neonatal convulsions) and perinatal mortality (ie, stillbirths and deaths) during the first 28 days of life. Multivariable generalized estimating equations with logit-link were used to obtain adjusted odds ratios (aORs) and 95% CIs.

**Results:**

The mean (SD) age at delivery for women in the epilepsy cohort was 29.9 (5.3) years. The rate of composite severe maternal morbidity and mortality was also higher in women with epilepsy compared with those without epilepsy (36.9 vs 25.4 per 1000 deliveries). Women with epilepsy also had a significantly higher risk of death (0.23 deaths per 1000 deliveries) compared with women without epilepsy (0.05 deaths per 1000 deliveries) with an aOR of 3.86 (95% CI, 1.48-8.10). In particular, maternal epilepsy was associated with increased odds of severe preeclampsia, embolism, disseminated intravascular coagulation or shock, cerebrovascular events, and severe mental health conditions. Fetuses and infants of women with epilepsy were at elevated odds of mortality (aOR, 1.20; 95% CI, 1.05-1.38) and severe neonatal morbidity (aOR, 1.48; 95% CI, 1.40-1.56). In analyses restricted to women with epilepsy, women exposed to ASM compared with those unexposed had higher odds of severe maternal morbidity (aOR ,1.24; 95% CI, 1.10-1.48) and their neonates had an increased odd of mortality and severe morbidity (aOR, 1.37; 95% CI, 1.23-1.52).

**Conclusion and relevance:**

This multinational study shows that women with epilepsy were at considerably higher risk of severe maternal and perinatal outcomes and increased risk of death during pregnancy and postpartum. Maternal epilepsy and maternal use of ASM were associated with increased maternal morbidity and perinatal mortality and morbidity.

## Key Points

**Question:**

What are the associations between maternal epilepsy, antiseizure medication use during pregnancy, and risks of severe maternal and perinatal morbidity and mortality?

**Findings:**

This multinational study that included 4 511 267 pregnancies showed that women with epilepsy were at considerably higher risk of severe maternal and perinatal outcomes and increased risk of death during pregnancy and postpartum. Maternal epilepsy and maternal use of antiseizure medication were associated with increased maternal morbidity and perinatal mortality and morbidity.

**Meaning:**

While most women with epilepsy have uncomplicated pregnancies, there is an urgent need for enhanced counselling, perinatal support, and access to specialized care for safe deliveries in all women with epilepsy.

## Teaser

This study attempts to determine the risk of severe maternal and perinatal morbidity and mortality among women with epilepsy.

## Introduction

Epilepsy is a common neurologic disorder affecting 65 million individuals worldwide and an estimated 0.5% to 1.0% of all pregnancies occur among women with epilepsy.^1,2^ Pregnancy complications among women with epilepsy extend beyond the effects of antiseizure medication (ASM) on the fetus and these complications can lead to substantial maternal and perinatal morbidity and mortality.^3,4,5,6^ In fact, women with epilepsy are at an increased risk of death in pregnancy and in the postpartum period.^7,8^

Severe maternal morbidity,^9^ which encompasses life-threatening and disabling conditions during pregnancy,^10^ has profound health implications for women.^11,12^ Few studies have shown that women with epilepsy face an elevated risk of specific severe maternal complications, such as eclampsia and placental abruption.^3,13,14,15^ Recognizing these obstetric challenges is imperative for preventing maternal mortality in this group. Additionally, other severe maternal conditions, including embolism, cardiac disorders, complications arising from obstetric procedures, and severe postpartum hemorrhage, can contribute to maternal mortality in women with epilepsy.^16^

Previous studies on epilepsy in pregnancy had small sample sizes and also lacked a comprehensive analysis of severe adverse fetal and infant outcomes. Additionally, robust evidence regarding the association between ASM use during pregnancy, which likely reflects more severe illness, and maternal and perinatal morbidity and mortality is limited.^13^ Therefore, we carried out a multinational study aimed at quantifying the risks of severe morbidity and mortality in women with epilepsy and their children. We also compared outcomes between women with epilepsy exposed and unexposed to ASM during pregnancy.

## Methods

### Data Sources and Study Population

This study was part of the SCAN-AED project, which aims to fill knowledge gaps in treatment guidelines for women needing ASM in pregnancy using the Nordic register infrastructure. Nordic countries each have a public funded health care system with universal coverage.^17^ We conducted a cohort study, including information on all singleton births at 22 or more completed gestational weeks in the 5 Nordic countries, namely, Denmark (1997 to 2017), Finland (1996 to 2016), Iceland (2004 to 2017), Norway (2005 to 2017), and Sweden (2006 to 2017). The Nordic registers from which data were obtained included complete longitudinal data on maternal medication history from at least 3 months before the last menstrual period (LMP) with regard to the pregnancy of interest. In this article, we refer to birthing parents as women, but we acknowledge that this group also includes gender-diverse people, as well as adolescent girls. The project received approval from the appropriate ethics and data protection authorities in each country. A waiver of informed consent was granted as register-based studies using pseudonymized data are exempt from consent requirements under Nordic data protection legislation. No participants were contacted or provided with compensation. This study followed the Strengthening the Reporting of Observational Studies in Epidemiology (STROBE) reporting guidelines.

We excluded singleton births with recorded gestational length of 314 days or longer, implausible combinations of birth weight and pregnancy length, and missing information on birth weight or gestational length and women who were only exposed to ASMs prior to pregnancy (in the 90 days before the LMP) (eFigure in Supplement 1). Using the person-unique national registration numbers of mothers and their offspring, we identified pregnancy characteristics, prescription fills, mother and child diagnoses, and demographic and socioeconomic information from the national health and social registers in each country. We harmonized variable definitions across the 5 countries based on a common data model^18^ (eTable 1 and eTable 2 in Supplement 1). All maternal and neonatal conditions were ascertained from each country’s Medical Birth Register, Patient Register, and Cause of Death Register records, according to the *International Statistical Classification of Diseases and Related Health Problems, Tenth Revision (ICD-10)*.^19^

### Exposure

#### Maternal Epilepsy and ASM Exposure

Maternal epilepsy was defined as one or more diagnosis codes for epilepsy (*ICD-10* codes G40 and G41) in the Patient Register or the Medical Birth Register up to 10 years prior to the LMP for the index pregnancy. In Norway, Denmark, and Finland, it also included 1 or more prescriptions for ASM with epilepsy as the reimbursement code (eTable 3 in Supplement 1). The epilepsy cohort was restricted to individuals whose epilepsy onset occurred before their child’s birth and those with active epilepsy (ie, diagnosis code for epilepsy within 10 years prior to conception).^20^ The validity and reliability of *ICD* codes for epilepsy in health registers are moderate to high.^21,22,23,24,25,26^ We defined mothers who filled a prescription for ASM using nationwide prescription registers and identified ASMs based on Anatomical Therapeutic Chemical Classification^27^ codes N03, N05BA09, and S01EC01. The exposure window for ASMs was defined as the period between the date of the LMP and the day of birth: women who filled a prescription within this window were considered exposed, while unexposed women included those who did not fill an ASM prescription in the period between 90 days before the LMP and the day of birth. Monotherapy referred to using only 1 type of ASM during the exposure period, while polytherapy was defined as receipt of 2 or more different ASMs (further stratified into combinations with and without valproate).

### Outcomes

The primary maternal outcome was a composite of severe maternal morbidity or mortality. Severe maternal morbidity included heterogeneous maternal conditions with a high-case fatality (eg, amniotic fluid embolism), organ failure (eg, acute kidney failure), or serious sequelae (eg, intracranial hemorrhage) and maternal death included death in pregnancy from 22 weeks’ or longer gestation to 42 days postpartum.

Severe maternal morbidity conditions were identified based on a comprehensive list of indicators (eTable 4 in Supplement 1), previously developed by the Canadian Perinatal Surveillance System^28^ and the US Centers for Disease Control and Prevention US,^29,30^ and recently modified and validated for use with the Nordic health register.^31^ In this study, we excluded conditions that are more prevalent among individuals with epilepsy, such as status epilepticus (G41) and shock (R57), to accurately capture severe maternal morbidity conditions and prevent over ascertainment. Severe maternal morbidity was categorized into specific groups including: (1) eclampsia or severe preeclampsia, (2) severe hemorrhage, (3) surgical complications, eg, disruption or hematoma of obstetric wound, (4) sepsis, eg, major puerperal infection, septicemia during labor, (5) obstetric shock, (6) cardiac complications, eg, acute myocardial infarction, heart failure, (7) kidney failure, (8) cerebrovascular morbidity, eg, cerebral venous thromboembolism, intracranial hemorrhage, (9) complications of anesthesia and obstetric interventions, including cardiac and pulmonary complications, and (10) severe mental health conditions, ie, suicide attempts, and any hospitalization for psychiatric disorder as a primary diagnosis (eTable 4 in Supplement 1). These categories were not mutually exclusive and each condition required at least 1 inpatient stay during pregnancy or within 42 days postpartum with the corresponding diagnostic codes listed as the primary or secondary diagnosis. The composite outcome of severe maternal morbidity included 1 or more of these conditions. For this study, maternal mortality, was defined as death (from cause of death) that occurred between 22 weeks’ gestation and 42 days postpartum.

The primary outcome in offspring was the composite perinatal outcome, including perinatal death or severe neonatal morbidity. Stillbirth was defined as antepartum or intrapartum fetal death with delivery at or after 22 weeks of gestation and neonatal death was defined as infant death within 28 days after birth. Extended perinatal mortality (hereafter referred to as perinatal mortality) was defined as stillbirth or neonatal death.^32^ Composite severe neonatal morbidity included intracranial hemorrhage, periventricular leukomalacia, neonatal convulsions, retinopathy of prematurity, respiratory distress syndrome, bronchopulmonary dysplasia, pneumothorax, necrotizing enterocolitis, hypoxic ischemic encephalopathy, perinatal intestinal perforation, sepsis, and severe birth trauma (eTable 4 in Supplement 1).

### Covariates and Additional Variables

Maternal characteristics assessed included age at delivery, parity, country of birth, education level, cohabitation with a partner, early or prepregnancy body mass index (BMI), smoking in early pregnancy, and year of delivery. We also studied prepregnancy maternal psychiatric comorbidity, including bipolar and personality disorder, and mood and anxiety disorders, as well as counts of somatic diagnoses and prepregnancy hospitalization before pregnancy as a measure of comorbidity (eTable 2 in Supplement 1).

### Statistical Analyses

Data were stored at Statistics Denmark and analyzed using Stata version 17.0 (Stata Corp) and RStudio (R Institute). We assessed the distribution of maternal characteristics by epilepsy status and ASM use among women with epilepsy. We used generalized estimating equations (GEE) with logit link, exchangeable correlation structure and robust standard errors to obtain odds ratios (ORs) and 95% CIs. In the multivariable analyses, we adjusted for maternal age, parity, birth year, child’s sex, mother’s education, marital status, country, maternal prepregnancy psychiatric comorbidity, number of somatic diagnoses, and hospitalizations in the year preceding pregnancy. Maternal and perinatal outcomes in women with epilepsy treated with ASMs during pregnancy (vs women with epilepsy not exposed to ASMs 3 months prior to pregnancy or during pregnancy) were examined using the same methodology mentioned above. No adjustments were made for multiple comparisons and the results of this study should be considered exploratory.

Except for maternal smoking status and BMI, which were not included in the main analysis, there were few missing values for all other covariates (ranging from 0 to 3%, as shown in Table 1). To handle missing data, we used multiple imputation with chained equations to create 20 datasets with imputed values for various factors, including parity, education, marital status, BMI, and smoking. Imputation methods involved linear regression for BMI, ordinal logistic regression for parity and education, and logistic regression for smoking and marital status. All variables in subsequent regression models, including composite outcomes for maternal and fetal/infant morbidity, were included in the imputation equations. We included binary variables for maternal foreign-born status and gestational diabetes as auxiliary variables for improved imputation accuracy. Gestational diabetes was included due to its strong association with BMI, while being born outside the 5 Nordic countries was associated both with BMI, smoking, and level of education. In Iceland, where smoking data was lacking (1.3% of total observations), we imputed smoking values based on assumed associations with other variables, considering it did not significantly impact results.

**Table 1.** Maternal Characteristics According to Maternal Epilepsy and Maternal Antiseizure Medications (ASM) Use During Pregnancy in 5 Nordic Countries, 1997 to 2017

Maternal characteristic	No. (%)			
	No epilepsy (n = 4 475 984)	Epilepsy (n = 35 283)	Epilepsy, no ASM (n = 19 043)	Epilepsy and ASM (n = 16 240)
Country				
Denmark	1 233 240 (99.1)	11 372 (0.9)	6721 (59.1)	4651 (40.9)
Finland	1 171 456 (99.5)	5841 (0.5)	1167 (20.0)	4674 (80.0)
Iceland	58 809 (99.6)	261 (0.4)	82 (31.4)	179 (68.6)
Norway	737 755 (98.8)	8590 (1.2)	5748 (66.9)	2842 (33.1)
Sweden	1 274 724 (99.3)	9219 (0.7)	5325 (57.8)	3894 (42.2)
Year of birth				
1996-1999	407 076 (99.6)	1432 (0.4)	379 (26.5)	1053 (73.5)
2000-2004	581 200 (99.4)	3448 (0.6)	1386 (40.2)	2062 (59.8)
2005-2009	1 297 200 (99.2)	10 572 (0.8)	5834 (55.2)	4738 (44.8)
2010-2014	1 406 422 (99.1)	12 754 (0.9)	7342 (57.6)	5412 (42.4)
2015-2017	784 086 (99.1)	7077 (0.9)	4102 (58.0)	2975 (42.0)
Maternal age, y mean (SD)	30.2 (5.2)	29.9 (5.3)	29.7 (5.4)	30.1 (5.2)
Maternal age, y				
<20	64 274 (1.4)	698 (2.0)	424 (2.2)	274 (1.7)
20-24	562 742 (12.6)	5214 (14.8)	3077 (16.2)	2137 (13.2)
25-29	1 385 096 (30.9)	10 851 (30.8)	5820 (30.6)	5031 (31.0)
30-34	1 525 710 (34.1)	11 383 (32.3)	5944 (31.2)	5439 (33.5)
≥35	938 057 (21.0)	7137 (20.2)	3778 (19.8)	3359 (20.7)
Missing	105 (0)	<5	0 (0)	<5
Parity				
0	1 924 542 (43.0)	15 867 (45.0)	8320 (43.7)	7547 (46.5)
1	1 601 657 (35.8)	12 170 (34.5)	6674 (35.0)	5496 (33.8)
≥2	926 812 (20.7)	7102 (20.1)	4000 (21.0)	3102 (19.1)
Missing	22 973 (0.5)	144 (0.4)	49 (0.3)	95 (0.6)
Education				
Compulsory	612 882 (13.7)	8304 (23.5)	4994 (26.2)	3310 (20.4)
Preuniversity	2 081 006 (46.5)	15 998 (45.3)	8167 (42.9)	7831 (48.2)
Bachelor	980 553 (21.9)	6746 (19.1)	3564 (18.7)	3182 (19.6)
Master/PhD	615 545 (13.8)	3170 (9.0)	1783 (9.4)	1387 (8.5)
Missing	185 998 (4.2)	1065 (3.0)	535 (2.8)	530 (3.3)
Married/cohabiting				
No	331 761 (7.4)	3846 (10.9)	2175 (11.4)	1671 (10.3)
Yes	4 063 027 (90.8)	30 993 (87.8)	16 652 (87.4)	14 341 (88.3)
Missing	81 196 (1.8)	444 (1.3)	216 (1.1)	228 (1.4)
Early prepregnancy BMI^a^				
<18.5	100 774 (2.3)	869 (2.5)	501 (2.6)	368 (2.3)
18.5-24.0	1 844 997 (41.2)	14 085 (39.9)	7814 (41.0)	6271 (38.6)
25-29.9	696 054 (15.6)	6412 (18.2)	3491 (18.3)	2921 (18.0)
≥30	375 129 (8.4)	4137 (11.7)	2308 (12.1)	1829 (11.3)
Missing	1 459 030 (32.6)	9780 (27.7)	4929 (25.9)	4851 (29.9)
Smoking in pregnancy				
No	3 628 355 (81.1)	26 867 (76.1)	14 374 (75.5)	12 493 (76.9)
Yes	486 772 (10.9)	5807 (16.5)	3294 (17.3)	2513 (15.5)
Missing	360 857 (8.1)	2609 (7.4)	1375 (7.2)	1234 (7.6)
Chronic somatic comorbidities				
0	4 250 467 (95.0)	32 191 (91.2)	17 011 (89.3)	15 180 (93.5)
1	216 087 (4.8)	2624 (7.4)	1628 (8.5)	996 (6.1)
≥2	9430 (0.2)	468 (1.3)	404 (2.1)	64 (0.4)
Prepregnancy hospitalizations^b^				
0	3 797 527 (84.8)	27 137 (76.9)	14 657 (77.0)	12 480 (76.8)
1	559 700 (12.5)	5617 (15.9)	3065 (16.1)	2552 (15.7)
≥2	118 757 (2.7)	2529 (7.2)	1321 (6.9)	1208 (7.4)
Psychiatric comorbidity				
Depression	32 612 (0.7)	881 (2.5)	502 (2.6)	379 (2.3)
Anxiety	45 423 (1.0)	1893 (5.4)	886 (4.7)	1007 (6.2)
Personality disorder	10 487 (0.2)	502 (1.4)	289 (1.5)	213 (1.3)
Bipolar disorder	5470 (0.1)	382 (1.1)	197 (1.0)	185 (1.1)
Any psychiatric comorbidity^c^	242 416 (5.4)	5281 (15.0)	3167 (16.6)	2114 (13.0)
Infant characteristics				
Sex				
Female	2 178 723 (48.7)	17 154 (48.6)	9281 (48.7)	7873 (48.5)
Male	2 297 261 (51.3)	18 129 (51.4)	9762 (51.3)	8367 (51.5)
Preterm birth (<37 wk)	213 560 (4.8)	2552 (7.2)	1370 (7.2)	1182 (7.3)
Chromosomal abnormalities	8222 (0.2)	92 (0.3)	50 (0.3)	42 (0.3)
Major congenital anomalies	199 224 (4.5)	2214 (6.3)	1123 (5.9)	1091 (6.7)

Abbreviations: BMI, body mass index; PhD, Doctor of Philosophy.

^a^ Calculated as weight in kilograms divided by height in meters squared.

^b^ Time window: last menstrual period − 365 to last menstrual period.

^c^ Defined as depression, anxiety, personality disorder, bipolar disorder, schizophrenia, or other delusional disorders or any use of antidepressants or antipsychotics.

We performed several sensitivity analyses. First, interaction analyses were performed to investigate whether the association between maternal epilepsy and rates of severe morbidity and mortality was different in women with psychiatric comorbidity vs those without psychiatric comorbidity. Second, estimates were further adjusted for maternal BMI and smoking in early pregnancy. Third, to identify potential heterogeneity across countries, we performed country-wise analyses of maternal epilepsy and ASM exposure with each of the composite outcomes. Fourth, given that eclampsia could be potentially misclassified in the setting of epilepsy, we removed eclampsia from the severe morbidity. Lastly, the maternal epilepsy-severe maternal morbidity association was re-examined after restricting having a recorded epilepsy diagnosis within 1 year before the date of conception or reported in the medical birth register. This approach aimed to capture women with more active disease and recent health care contact prior to pregnancy.

## Results

The study team identified 4 511 267 deliveries of which 4 475 984 were to women without epilepsy and 35 283 to mothers with epilepsy. The mean (SD) age at delivery for women in the epilepsy cohort was 29.9 (5.3) years. Among deliveries to women with epilepsy, 16 240 offspring were exposed to ASM during pregnancy (46%). Women with epilepsy were more often younger, nulliparous, lived alone, had lower education, smoked during pregnancy, were obese, had more somatic and psychiatric comorbidity, and had more hospitalizations in the year preceding pregnancy than women without epilepsy (Table 1). Among women with epilepsy, those taking ASM during pregnancy were more often nulliparous and had a higher frequency of anxiety disorders. Offspring of women with epilepsy were more likely to be born preterm and have a major congenital anomaly.

The rate of composite severe maternal morbidity was higher in women with epilepsy compared with those without epilepsy (36.9 vs 25.3 per 1000 deliveries; Table 2). Women with epilepsy also had a significantly higher risk of death during delivery (2.3 deaths per 10 000 pregnancies) compared with women without epilepsy (0.4 deaths per 10 000 pregnancies; Table 2). Among specific conditions considered within severe maternal morbidity, rates of severe preeclampsia, hemorrhage, embolism, sepsis, cerebrovascular events, surgical complications, and severe maternal health were considerably higher in women with epilepsy (Table 2). After adjusting for maternal characteristics and prepregnancy factors, pregnancies in women with epilepsy were associated with higher rates of maternal death (adjusted odds ratio [aOR], 3.86; 95% CI, 1.84-8.10) and higher rates of severe maternal morbidity (aOR, 1.23; 95% CI, 1.16-1.31; Figure) compared with women without epilepsy. Odds of severe preeclampsia, hemolysis, elevated liver enzymes and low platelets (HELLP) syndrome, or eclampsia (aOR, 1.30; 95% CI, 1.19-1.42), embolism, disseminated intravascular coagulation, or shock (aOR, 1.42; 95% CI, 1.05-1.93), cerebrovascular events (aOR, 5.81; 95% CI, 4.27-7.89), and severe mental health conditions (aOR, 1.81; 95% CI, 1.50-2.19) were particularly elevated among women with epilepsy, compared with women without epilepsy (Figure).

**Table 2.** Maternal and Perinatal Mortality and Severe Morbidity by Maternal Epilepsy Status in 5 Nordic Countries, 1997 to 2017

Outcomes	Without epilepsy (n = 4 475 984)		With epilepsy (n = 35 283)		Unadjusted odds ratio (95% CI)^a^
	No. of events	Rate per 1000 deliveries/births (95% CI)	No. of events	Rate per 1000 deliveries/births (95% CI)	
Maternal morbidity and mortality outcome					
Composite maternal mortality/maternal morbidity^b^	112 115	25.4 (25.2-25.5)	1291	36.9 (34.9-38.9)	1.46 (1.38-1.55)
Maternal mortality^b^	206	0.05 (0.04-0.05)	8	0.23 (0.11-0.46)	4.90 (2.42-9.93)
Composite severe maternal morbidity	113 355	25.3 (25.2-25.5)	1298	36.9 (34.9-38.8)	1.46 (1.38-1.55)
Severe maternal morbidity					
Severe preeclampsia, HELLP, eclampsia	47 345	10.6 (10.5-10.7)	559	15.8 (14.6-17.2)	1.50 (1.37-1.63)
Severe hemorrhage	28 536	6.38 (6.30-6.45)	260	7.37 (6.53-8.32)	1.15 (1.02-1.30)
Pulmonary and obstetric embolism, DIC, shock	3258	0.73 (0.70-0.75)	44	1.25 (0.93-1.68)	1.71 (1.26-2.32)
Sepsis	22 715	5.07 (5.01-5.14)	228	6.46 (5.68-7.35)	1.27 (1.12-1.45)
Acute kidney failure	483	0.11 (0.10-0.12)	7	0.20 (0.09-0.42)	1.83 (0.87-3.88)
Cardiac complications	1201	0.27 (0.25-0.28)	17	0.48 (0.30-0.77)	1.80 (1.11-2.90)
Complications of anesthesia	429	0.10 (0.09-0.11)	<5	NA^c^	NA^c^
Cerebrovascular accidents	974	0.22 (0.20-0.23)	53	1.50 (1.15-1.97)	6.69 (4.96-9.02)
Surgical complications	3683	0.82 (0.80-0.85)	42	1.19 (0.88-1.61)	1.45 (1.07-1.96)
Severe mental health conditions	4576	1.02 (0.99-1.05)	117	3.32 (2.77-3.97)	3.21 (2.66-3.87)
Uterine rupture	4933	1.10 (1.07-1.13)	42	1.19 (0.88-1.61)	1.08 (0.79-1.47)
Fetal/infant mortality and morbidity outcome^d^					
Composite perinatal mortality/severe neonatal morbidity^e^	132 990	29.7 (29.6-29.9)	1672	47.4 (45.2-49.7)	1.62 (1.54-1.70)
Perinatal mortality					
Stillbirth (after 22 wk gestation)^f^	13 975	3.12 (3.07-3.17)	138	3.91 (3.31-4.62)	1.25 (1.06-1.48)
Neonatal death (0-27 d)^g^	7035	1.58 (1.54-1.61)	75	2.13 (1.70-2.68)	1.36 (1.08-1.70)
Perinatal mortality (stillbirth and neonatal death)^f^	21 010	4.69 (4.63-4.76)	213	6.04 (5.28-6.90)	1.29 (1.12-1.47)
Composite severe neonatal morbidity^e^^,^^g^	114 665	25.7 (25.6-25.9)	1496	42.6 (40.5-44.7)	1.68 (1.59-1.77)

Abbreviations: DIC, disseminated intravascular coagulation; HELLP, hemolysis, elevated liver enzymes and low platelets; NA, not applicable.

^a^ Odds ratios are exponentiated coefficients from generalized estimating equations model with binomial distribution, logit-link, and robust standard errors.

^b^ Data from Iceland excluded from analyses where mortality is included in the outcome because of lack of data on maternal mortality.

^c^ Owing to personal data protection restriction on publishing cell counts less than 5.

^d^ Stillborn children excluded from analyses of neonatal death and morbidity end points.

^e^ Includes respiratory distress syndrome, retinopathy of prematurity, intraventricular hemorrhage (grade 3 or higher), intracranial hemorrhage, sepsis, necrotizing enterocolitis, severe birth trauma, and seizures.

^f^ Stillbirths, perinatal deaths, and composite perinatal death/severe neonatal morbidity are expressed per 1000 total births.

^g^ Neonatal deaths and composite severe neonatal morbidity are expressed per 1000 live births.

**Figure.**
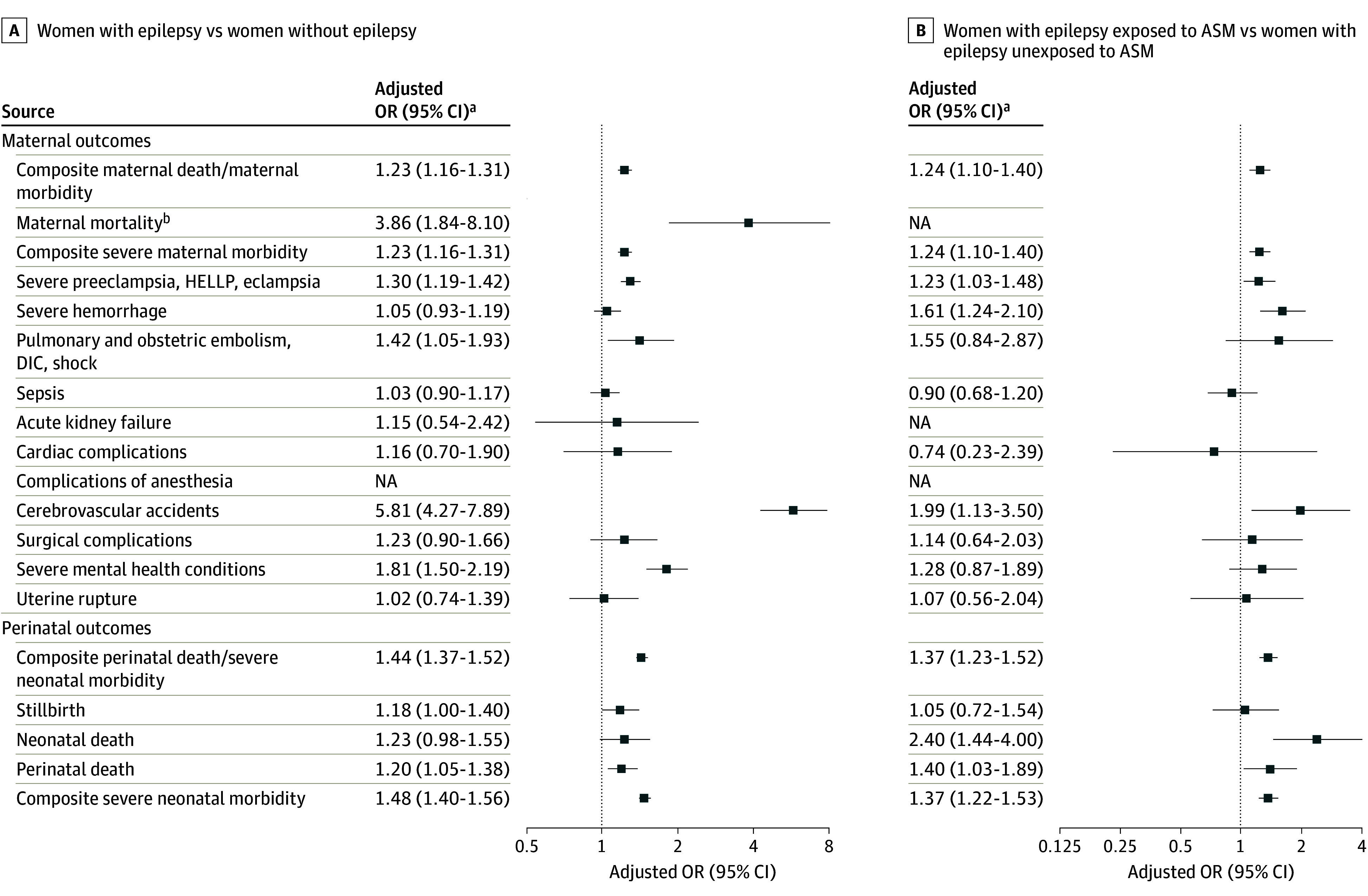
Adjusted Odds Ratios (ORs) of Severe Maternal and Perinatal Morbidity and Mortality Among Mothers With or Without Epilepsy and Among Women With Epilepsy by Antiseizure Medications (ASM) Use During Pregnancy in 5 Nordic Countries, 1997 to 2017 HELLP indicates hemolysis, elevated liver enzymes and low platelets; NA, not applicable. ^a^Adjusted for maternal age, parity, birth year, child’s sex mother’s education, marital status, country, maternal psychiatric morbidity, number of chronic conditions, and number of prepregnancy hospitalizations. ^b^Data from Iceland excluded from analyses where mortality is included in the outcome because of lack of data on maternal mortality.

Rates of composite perinatal death/severe neonatal morbidity were higher among offspring of women with epilepsy compared with offspring of women without epilepsy (0.47 vs 0.3 per 1000 births; Table 2). After adjusting for potential confounders, women with epilepsy had a 1.18-fold higher odds of stillbirth (95% CI, 1.00-1.40), a 1.23-fold higher odds of neonatal death (95% CI, 0.98-1.55), and a 1.48-fold higher odds of composite severe neonatal morbidity (95% CI, 1.40-1.56), compared with women without epilepsy (Figure). Adjusted rates of hypoxic ischemic encephalopathy, neonatal convulsions, respiratory distress syndrome, and retinopathy of prematurity were substantially higher among neonates of women with epilepsy than those without epilepsy (eTable 5 in Supplement 1).

Among women with epilepsy, those exposed to ASM had higher rates (Table 3) and odds of composite severe maternal morbidity compared with deliveries to women who were not exposed to ASM (aOR, 1.24; 95% CI, 1.10-1.40; Figure). Women with epilepsy exposed to ASM during pregnancy had higher odds of severe preeclampsia, severe hemorrhage, and cerebrovascular events compared with women with epilepsy who were not exposed. Among 16 240 women exposed to ASM in monotherapy, lamotrigine (n = 5080 [31%]), carbamazepine (n = 2628 [16%]), and valproate (n = 1895 [12%]) were the most common ASMs (Table 4). Among women with epilepsy exposed to ASM in monotherapy vs no ASM, the odds of severe maternal morbidity were increased for valproate (aOR, 1.67; 95% CI, 1.26-2.23), carbamazepine (aOR, 1.46; 95% CI, 1.14-1.87), and oxcarbazepine (aOR, 1.53; 95% CI, 1.08-2.17; Table 4).

**Table 3.** Maternal and Perinatal Mortality and Severe Morbidity Among Women With Epilepsy by Antiseizure Medications (ASM) Use During Pregnancies in 5 Nordic Countries (1997 to 2017)

Outcome	No ASM (n = 19 013)		Any ASM (n = 16 240)		Unadjusted odds ratio (95% CI)^a^^,^^b^
	No. of events	Rate per 1000 deliveries/births (95% CI)	No. of events	Rate per 1000 deliveries/births (95% CI)	
Maternal morbidity and mortality outcome					
Composite maternal mortality/maternal morbidity^c^	655	34.6 (32.1-37.3)	636	39.6 (36.7-42.8)	1.16 (1.04-1.30)
Maternal mortality^c^	<5	NA^d^	<5	NA^d^	NA^d^
Composite severe maternal morbidity	656	34.5 (32.0-37.2)	642	39.6 (36.7-42.7)	1.17 (1.04-1.30)
Severe maternal morbidity					
Severe preeclampsia, HELLP, eclampsia	278	14.6 (13.0-16.4)	281	17.3 (15.4-19.4)	1.20 (1.01-1.43)
Severe hemorrhage	123	6.5 (5.4-7.7)	137	8.4 (7.1-10.0)	1.31 (1.02-1.67)
Pulmonary and obstetric embolism, DIC, shock	20	1.1 (0.7-1.6)	24	1.5 (1.0-2.2)	1.42 (0.77-2.61)
Sepsis	137	7.2 (6.1-8.5)	91	5.6 (4.6-6.9)	0.78 (0.60-1.02)
Acute kidney failure	<5	NA^d^	<5	NA^d^	NA^d^
Cardiac complications	11	0.6 (0.3-1.0)	6	0.4 (0.2-0.8)	0.64 (0.24-1.73)
Complications of anesthesia	<5	NA^d^	<5	NA^d^	NA^d^
Cerebrovascular accidents	19	1.0 (0.6-1.6)	34	2.1 (1.5-2.9)	2.15 (1.21-3.82)
Surgical complications	22	1.0 (0.6-1.6)	20	2.1 (1.5-2.9)	2.15 (1.21-3.82)
Severe mental health conditions	56	2.9 (2.3-3.8)	61	3.8 (2.9-4.8)	1.28 (0.89-1.85)
Uterine rupture	21	1.1 (0.7-1.7)	21	1.3 (0.8-2.0)	1.16 (0.62-2.17)
Fetal/infant mortality and morbidity outcome^e^					
Composite perinatal death/severe neonatal morbidity^d^	760	39.9 (37.2-42.8)	912	56.2 (52.7-59.8)	1.17 (0.84-1.64)
Perinatal death^f^					
Stillbirth^f^	69	3.62 (2.86-4.59)	69	4.25 (3.36-5.38)	2.65 (1.62-4.33)
Neonatal death^g^	23	1.21 (0.81-1.82)	52	3.22 (2.45-4.22)	1.55 (1.18-2.03)
Perinatal death^f^	92	4.83 (3.94-5.92)	121	7.45 (6.24-8.90)	1.42 (1.28-1.58)
Composite severe neonatal morbidity^d^^,^^g^	684	36.1 (33.5-38.8)	812	50.2 (47.0-53.7)	1.42 (1.28-1.58)

Abbreviations: DIC, disseminated intravascular coagulation; HELLP, hemolysis, elevated liver enzymes and low platelets; NA, not applicable.

^a^ Odds ratios are exponentiated coefficients from generalized estimating equations model with binomial distribution, logit-link, and robust standard errors.

^b^ Adjusted for maternal age, parity, birth year, child’s gender, mother’s education, marital status, country, and count of chronic comorbidities.

^c^ Data from Iceland excluded from analyses where mortality is included in the outcome because of lack of data on maternal mortality.

^d^ Owing to personal data protection restriction on publishing cell counts less than 5.

^e^ Stillborn children excluded from analyses of neonatal death and morbidity end points.

^f^ Includes respiratory distress syndrome, retinopathy of prematurity, intraventricular hemorrhage (grade 3 or higher), intracranial hemorrhage, sepsis, necrotizing enterocolitis, severe birth trauma, and seizures.

^g^ Neonatal deaths and composite severe neonatal morbidity are expressed per 1000 live births.

**Table 4.** Severe Maternal and Perinatal Morbidity and Mortality (per 1000 Pregnancies) Among Women With Epilepsy by Antiseizure Medication (ASM) Monotherapy and Polytherapy During Pregnancy in 5 Nordic Countries (1997 to 2017)

Exposure	No. of pregnancies	Composite severe maternal morbidity		Composite perinatal death or severe neonatal morbidity	
		No. of events (rate per 1000 deliveries/births)	Adjusted OR^a^ (95% CI)^b^	No. of events (rate per 1000 deliveries/births)	Adjusted OR^a^ (95% CI)^b^
No ASM use	19 043	656 (34.4)	1 [Reference]	760 (39.9)	1 [Reference]
Any ASM^c^	16 240	642 (39.5)	1.24 (1.10-1.40)	912 (56.2)	1.37 (1.23-1.52)
Monotherapy					
Lamotrigine	5080	174 (34.2)	0.99 (0.83-1.18)	223 (43.9)	1.14 (0.97-1.33)
Carbamazepine	2628	103 (39.2)	1.46 (1.14-1.87)	133 (50.6)	1.32 (1.04-1.67)
Valproate	1895	78 (41.2)	1.67 (1.26-2.23)	142 (74.9)	1.85 (1.47-2.33)
Pregabalin	89	7 (78.7)	1.41 (0.66-3.03)	12 (134)	2.46 (1.31-4.61)
Gabapentin	108	<5	NA^d^	10 (92.6)	1.94 (0.98-3.84)
Oxcarbazepine	1439	56 (38.9)	1.53 (1.08-2.17)	64 (44.5)	0.99 (0.73-1.35)
Clonazepam	321	10 (31.2)	0.91 (0.48-1.72)	44 (137)	3.14 (2.21-4.46)
Levetiracetam	1009	45 (44.6)	1.27 (0.93-1.75)	40 (39.6)	1.03 (0.73-1.44)
Topiramate	249	10 (40.2)	1.21 (0.64-2.31)	13 (52.2)	1.23 (0.71-2.13)
Phenobarbital	45	<5	NA^d^	<5	NA^d^
Any monotherapy	13 039	494 (37.9)	1.22 (1.07-1.39)	689 (52.8)	1.32 (1.17-1.48)
Polytherapy					
With valproate	935	39 (41.7)	1.29 (0.91-1.85)	89 (95.2)	2.21 (1.70-2.87)
Without valproate	2266	109 (48.1)	1.36 (1.09-1.70)	134 (59.1)	1.31 (1.07-1.61)
Any polytherapy	3201	148 (46.2)	1.33 (1.09-1.62)	223 (69.7)	1.55 (1.31-1.84)

Abbreviations: NA, not applicable; OR, odds ratio.

^a^ ORs are exponentiated coefficients from generalized estimating equations model with binomial distribution, logit-link, and robust standard errors.

^b^ Adjusted for maternal age, parity, birth year, child’s sex, mother’s education, marital status, country, maternal psychiatric morbidity, number of chronic conditions, and number of prepregnancy hospitalizations. The reference category for each monotherapy is no ASM use.

^c^ Numbers of monotherapy and polytherapy do not add up to any ASM exposure because the following monotherapies are not included because of low numbers: gabapantin, phenobarbital.

^d^ Owing to personal data protection restriction on publishing cell counts less than 5.

Fetuses and infants of women with epilepsy exposed to ASM had higher rates of perinatal death/severe neonatal morbidity (composite outcome 56.2 vs 39.9 per 1000 total births), compared with fetuses and infants of women with epilepsy not exposed to ASM (Table 3). In the adjusted models, neonates of women with epilepsy exposed to ASM during pregnancy had elevated odds of neonatal death (aOR, 2.40; 95% CI, 1.44-3.99) and composite severe neonatal morbidity (aOR, 1.37; 95% CI, 1.23-1.52; Table 3; Figure). These neonates had increased odds of hypoxic ischemic encephalopathy, respiratory distress syndrome, sepsis, and severe birth trauma (eTable 4 in Supplement 1). Exposure to clonazepam, pregabalin, gabapentin, valproate, and carbamazepine was associated with an increased odds of perinatal death/severe neonatal morbidity (Table 4). Compared with neonates not exposed to ASM, those exposed to polytherapy with valproate has a 2.21-fold higher odds of perinatal mortality and severe morbidity (95% CI, 1.70-2.87).

In the sensitivity analyses, stratified analyses by maternal psychiatric comorbidity showed similar findings with a higher rate of composite perinatal death/severe neonatal morbidity among women with epilepsy and psychiatric comorbidity (eTable 6 in Supplement 1). Repeating the primary analyses after adjusting for maternal BMI, smoking, number of somatic diagnoses, and hospitalizations in the year preceding pregnancy did not change the findings (eTable 7 and eTable 8 in Supplement 1). Country-specific analyses of association between maternal epilepsy and the composite severe maternal morbidity and mortality end point showed small variations in absolute rates of severe maternal and perinatal morbidity and mortality but there was substantial overlap between the 95% CIs of the effect estimates (eTable 9 in Supplement 1). Excluding eclampsia from the severe preeclampsia/HELLP/eclampsia outcome did not impact the findings (eTable 10 in Supplement 1). Restricting maternal epilepsy to women with a diagnosis within 1 year preceding pregnancy strengthened the association (eTable 11 in Supplement 1).

## Discussion

In this large multinational cohort including over 4.5 million deliveries, we found that women with epilepsy had a 23% higher risk of life-threatening complications and approximately 4-fold higher risk of death and in pregnancy and the postpartum period. In addition, fetuses and infants of women with epilepsy had a 20% higher risk of perinatal death and 50% increased risk of severe neonatal morbidity compared with offspring of women without epilepsy. Specifically, women with epilepsy were at elevated risk of experiencing severe preeclampsia, HELLP syndrome or eclampsia, embolism, cerebrovascular events, and severe mental health conditions, while the fetuses and infants of these women has elevated risks of hypoxic ischemic encephalopathy, neonatal convulsions, respiratory distress syndrome, and retinopathy of prematurity. Exposure to ASM during pregnancy was also associated with increase in risks of severe maternal and perinatal morbidity and mortality.

Our study showed an elevated risk of maternal mortality in women with epilepsy, even in high-income Nordic countries with universal health care. Epilepsy is the second most common indirect cause of maternal death in the United Kingdom and Ireland and women with epilepsy have up to 10 times higher risk compared with women without epilepsy.^3,33^ A recent systematic review and meta-analysis^7^ reported that women with epilepsy have a 5 times higher odds of maternal death. Among women with epilepsy, other studies have shown that sudden unexpected death in epilepsy is one of the leading causes of maternal death.^4,16^In our data, we show that this patient group is also at risk of several other life-threatening disorders. The higher risks of maternal mortality underscore the urgent need for increased vigilance and comprehensive care throughout pregnancy and the postpartum period for women with epilepsy. Future studies should examine the pathways leading to death and the underlying causes of death in this population.

Our study found a 3- to 5-fold higher risk of severe maternal morbidity in women with epilepsy, including higher rates of severe preeclampsia/eclampsia, severe antepartum and postpartum hemorrhage,^3^ cerebrovascular complications,^13^ acute respiratory distress syndrome, and acute kidney failure.^14^ Our results show higher risks of cerebrovascular complications, including stroke among pregnant women with epilepsy, which is consistent with the literature showing a 3-fold higher incidence of stroke among younger epilepsy patients taking high doses of ASM.^34,35^ Life-threatening obstetric complications, including pulmonary embolism, cerebrovascular events, and cerebral vein thrombosis, likely contribute to the heightened risk of maternal death in women with epilepsy.^3,16^ Although most women with epilepsy (approximately 96%) will not experience severe adverse maternal and perinatal outcomes, the increased risks of severe complications during pregnancy may be due to the comorbidity associated with epilepsy, including psychiatric comorbidity, in addition to seizure-related factors.^36,37^ While we attempted to control for somatic and psychiatric comorbidities, it is unlikely that all aspects of human behavior and contributing factors, such as medication adherence, were captured using our register data, potentially influencing these severe outcomes.

In line with previous studies,^38,39^ our findings revealed elevated risks of hospitalization for psychiatric disorders in pregnancy and within 42 days post partum among women with epilepsy. This finding is concerning due to the high prevalence of psychiatric comorbidity in patients with epilepsy.^40^ Given that 25% of pregnancy-related deaths in the general population involves mental health conditions, including suicide,^41,42^ public health initiatives are required to enhance screening and treatment of perinatal mental health conditions in women with epilepsy.

We found that women with epilepsy who received ASM during pregnancy had increased risk of severe maternal morbidity, which likely reflects confounding by indication since those receiving treatment likely have severe epilepsy.^43,44^ Thus, ASM use can serve as a marker that identifies patients at higher risk of severe morbidity/mortality. Another study similarly found a 3-fold increased risk of severe maternal morbidity among women with epilepsy receiving ASM compared with women without epilepsy.^15^

We noted a 21% increased risk of perinatal death among women with epilepsy and a 50% increased risk of perinatal death among those receiving ASM during pregnancy. This magnitude of increased risk is consistent with that found in previous studies.^5,7^ Maternal exposure to epilepsy and ASM have been associated with a range of adverse offspring outcomes, including an increased risk of dying in infancy,^6^ autism disorders, and intellectual disability.^45^ Moreover, similar to previous studies,^5,7,46^ we found that infants of women with epilepsy were more likely than those without to experience severe neonatal morbidity, including hypoxic ischemic encephalopathy, neonatal convulsions, respiratory distress syndrome, and retinopathy of prematurity.^6^

### Strengths and Limitations

To our knowledge, this is the largest study on the association between maternal epilepsy (and prenatal exposure to ASM) with mortality and other clinically significant maternal and perinatal complications. We used nationwide and mandatory registry data from 5 Nordic counties. Our study has a few limitations. First, we relied on ASM prescription fills and compliance with treatment was unknown. However, previous research has shown high agreement between filled prescriptions and maternal reporting of ASM use during pregnancy.^47^ Second, we conducted a number of analyses without adjusting for multiple comparisons; therefore, results should be considered exploratory. Third, country-specific variations in data availability and diagnostic practices exist. For instance, data on maternal mortality were not available in Iceland and the length of look-back in the registers to identify maternal epilepsy varied, with only 1 year before pregnancy in Iceland and Finland compared with up to 10 years in Denmark and Sweden. However, there was very little variation in the study findings between countries. Fourth, we lacked information on seizure frequency and epilepsy control during pregnancy, which would have added to the clinical relevance of our findings. Fifth, we excluded multiple births due to their higher rates of maternal and perinatal complications and because birth outcomes among such pregnancies are nonindependent. Sixth, we did not differentiate between various epilepsy types due to data limitations, despite potential differences in their impact on pregnancy outcomes. Seventh, our data only cover pregnancies ending in delivery from 22 weeks’ gestation onwards, potentially underestimating maternal and other risks associated with epilepsy and ASM use in early gestation. Lastly, despite adjusting for several comorbidities (both somatic and psychiatric), residual confounding by unmeasured conditions remains possible and the increased risk of maternal mortality observed in our study may be due to comorbidities linked to epilepsy. Future studies with larger sample sizes should examine this association further.

## Conclusions

In this multinational study, maternal epilepsy was associated with an increased risk of maternal and perinatal mortality and severe morbidity. Treatment with ASM in pregnancy in women with epilepsy was associated with elevated risks of morbidity/mortality in women and their offspring compared with women not receiving ASM, likely reflecting the greater severity of epilepsy in the former group. Although most women with epilepsy have uncomplicated pregnancies and are considered healthier compared with nonpregnant women with epilepsy, there is an urgent need for enhanced prepregnancy counseling, close monitoring and perinatal support, and access to specialized care for safe deliveries in all women with epilepsy.

## Notes

Supplement 1. eFigure 1 Flow chart of included and excluded births. eTable 1: Data sources eTable 2. List of variables and definitions eTable 4. Severe maternal and neonatal morbidity, ICD-10 codes. eTable 5. Severe neonatal Morbidity (per 1000 Births) by Maternal Epilepsy Status and drug exposure in Five Nordic Countries (1997-2017) eTable 6. Composite severe maternal and neonatal mortality and morbidity by maternal Epilepsy Status and drug exposure stratified by maternal psychiatric comorbidity in Five Nordic Countries (1997-2017) eTable 7. Maternal and Perinatal Mortality and Severe Morbidity by Maternal Epilepsy Status in Five Nordic Countries adjusting for adjusting for maternal BMI and smoking in early pregnancy (1997-2017) eTable 8. Maternal and Perinatal Mortality and Severe Morbidity Among Women with Epilepsy by Antiseizure Medications (ASMs) Use During Pregnancies adjusting for maternal BMI and smoking in early pregnancy (1997-2017) eTable 9: Maternal and perinatal severe morbidity by maternal epilepsy status and ASM exposure, stratified by country eTable 10: Maternal severe preeclampsia or HELLP (excluding eclampsia) eTable 11: Composite severe maternal and neonatal mortality and morbidity by maternal Epilepsy Status, restricting the epilepsy diagnosis within 1 year before the date of conception

Supplement 2. Data sharing statement
