# Supplementary material for: Risk of Perinatal and Maternal Morbidity and Mortality Among Pregnant Women With Epilepsy
Source: JAMA Neurol. 2024 Aug 5;81(9):985–95. doi: 10.1001/jamaneurol.2024.2375 (PMC11385047; doi:10.1001/jamaneurol.2024.2375)
Supplement: Supplement 1. — eFigure 1 Flow chart of included and excluded births. eTable 1: Data sources eTable 2. List of variables and definitions eTable 4. Severe maternal and neonatal morbidity, ICD-10 codes. eTable 5. Severe neonatal Morbidity (per 1000 Births) by Maternal Epilepsy Status and drug exposure in Five Nordic Countries (1997-2017) eTable 6. Composite severe maternal and neonatal mortality and morbidity by maternal Epilepsy Status and drug exposure stratified by maternal psychiatric comorbidity in Five Nordic Countries (1997-2017) eTable 7. Maternal and Perinatal Mortality and Severe Morbidity by Maternal Epilepsy Status in Five Nordic Countries adjusting for adjusting for maternal BMI and smoking in early pregnancy (1997-2017) eTable 8. Maternal and Perinatal Mortality and Severe Morbidity Among Women with Epilepsy by Antiseizure Medications (ASMs) Use During Pregnancies adjusting for maternal BMI and smoking in early pregnancy (1997-2017) eTable 9: Maternal and perinatal severe morbidity by maternal epilepsy status and ASM exposure, stratified by country eTable 10: Maternal severe preeclampsia or HELLP (excluding eclampsia) eTable 11: Composite severe maternal and neonatal mortality and morbidity by maternal Epilepsy Status, restricting the epilepsy diagnosis within 1 year before the date of conception [file jamaneurol-e242375-s001.pdf]

Supplemental Online Content

Razaz N, Igland J, Bjørk MH, et al. Risk of perinatal and maternal morbidity and mortality among pregnant women with epilepsy. *JAMA Neurol*. Published online August 5, 2024.
doi:10.1001/jamaneurol.2024.2375

eFigure 1. Flow chart of included and excluded births

eTable 1. Data sources

eTable 2. List of variables and definitions

eTable 4. Severe maternal and neonatal morbidity, ICD-10 codes

eTable 5. Severe neonatal Morbidity (per 1000 Births) by Maternal Epilepsy Status and drug exposure in Five Nordic Countries (1997-2017)

eTable 6. Composite severe maternal and neonatal mortality and morbidity by maternal Epilepsy Status and drug exposure stratified by maternal psychiatric comorbidity in Five Nordic Countries (1997-2017)

eTable 7. Maternal and Perinatal Mortality and Severe Morbidity by Maternal Epilepsy Status in Five Nordic Countries adjusting for adjusting for maternal BMI and smoking in early pregnancy (1997-2017)

eTable 8. Maternal and Perinatal Mortality and Severe Morbidity Among Women with Epilepsy by Antiseizure Medications (ASMs) Use During Pregnancies adjusting for maternal BMI and smoking in early pregnancy (1997-2017)

eTable 9. Maternal and perinatal severe morbidity by maternal epilepsy status and ASM exposure, stratified by country

eTable 10. Maternal severe preeclampsia or HELLP (excluding eclampsia)

eTable 11. Composite severe maternal and neonatal mortality and morbidity by maternal Epilepsy Status, restricting the epilepsy diagnosis within 1 year before the date of conception

This supplemental material has been provided by the authors to give readers additional information about their work.

© 2024 Razaz N et al. *JAMA Neurol.*

eFigure 1 Flow chart of included and excluded births.

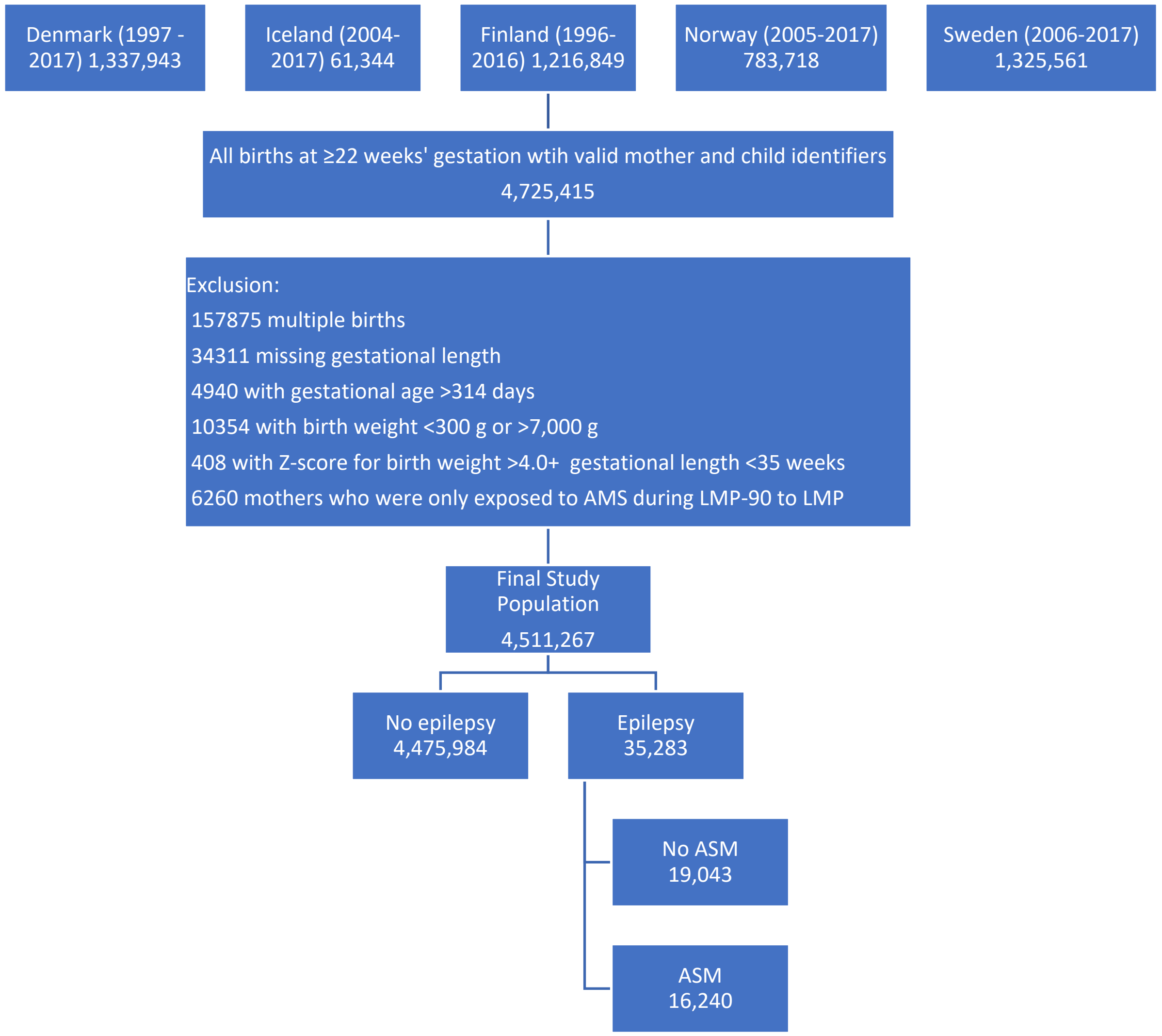

eTable 1: Data sources

© 2024 Razaz N et al. *JAMA Neurol.*

Registry	Availability	Description
Medical Birth Registry (MBR)	Denmark 1997<br>Finland 1996<br>Iceland 2003<br>Norway 2004<br>Sweden 2005	Information on births and stillbirths, term data, pregnancy length, maternal parity, smoking, body mass index, maternal age, maternal comorbidity, and pregnancy- and birth complications. Reporting is compulsory for maternal- and neonatal wards.<br>*Not available in Iceland.
Prescription drug registry (PDR)	Denmark 1996<br>Finland 1995[a]<br>Iceland 2003<br>Norway 2004<br>Sweden 2005	Data on all prescription fills from pharmacies including Anatomical therapeutical chemical (ATC) classification codes, drug strength, defined daily doses (DDD)[1] in a package, package size and dispensing date. Indication for use (reimbursement codes) available in Norway, Denmark and Finland. Over the counter medicines and medicines used during in-patient hospital stays not included. [a] In Finland, information is available only for all prescriptions entitled to reimbursement, while information on all medicines (irrespective of reimbursement) available in Denmark, Iceland, Norway, and Sweden.
National patient registry (NPR)	Denmark 1995[a]<br>Finland 1995[b]<br>Iceland 2003<br>Norway 2008<br>Sweden 2005[c]	Health administrative registries from public specialist care. Data on inpatient stays and outpatient care contacts included diagnoses coded according to International Statistical Classification of Diseases and Related Health Problems revision 10 (ICD-10)[2].<br>[a] From 1995 data from outpatient contacts and psychiatric treatment is included. [b] From 1998 data from outpatient visits in public hospitals<br>[c] Since 2001 outpatient visits (including psychiatric care) from private and public caregivers
National statistical institutes (NSI)	Cover all years included in the study	Socio-economic and demographic data on study participants held by Statistics Denmark, Statistics Norway, Statistics Finland, Statistics Sweden and Statistics Iceland. Information on education, demographic data on emigration and death.

REFERENCE:

1. Defined Daily Dose (DDD). 2021. at https://www.who.int/tools/atc-ddd-toolkit/about-ddd.)
2. World_Health_Organization. The ICD-10 classification of mental and behavioural disorders: diagnostic criteria for research: World Health Organization; 1993.

© 2024 Razaz N et al. *JAMA Neurol.*

eTable 2. List of variables and definitions

				Codes	
**Cohort Characteristic**	**Time Frame**	**Functional Form of Variable and Comments**	**Registers Used**	**ATC**	**ICD-10**
First day of last menstrual period (LMP)	n/a	Date of first day in last menstrual period estimated as date of birth subtracted from gestational age at birth.	MBR		
Maternal age	At birth	Continuous.	MBR		
Country	At birth	Country of birth. DK, FI, IS, NO, SE.	MBR		
Marital status	Year of birth	Categorical: Married/cohabitant or not.	MBR		
Education	Year of birth	Ordinal (Compulsory, Secondary/Pre-university, Bachelor, Master/PhD) categorized according to International standard classification for education (ISCED)	NSI		
Calendar year of birth	Year of birth	Continuous.	MBR		
Parity	n/a	Number of previous deliveries: 0,1, or ≥2	MBR		
Child sex	n/a	Categorical: Female or male.	MBR		
Pre-pregnancy admittances	LMP-365 to LMP	Categorical. Number of pre-pregnancy hospital admittances: 0, 1, or ≥2	NPR		
**Somatic comorbidities for the mother**					
Asthma	LMP-365 to LMP	Categorical: Y/N. At least one registration in NPR or registered in MBR at time of birth. FI: includes reimbursement codes	NPR MBR		J45 J46
Hypertension	LMP-365 to LMP	Categorical: Y/N.<br>Pre-gestational hypertension	NPR MBR		I10 I13 I15 O10.0 O10.3 O10.4, O10.9
Renal disease	LMP-365 to LMP	Categorical: Y/N.	NPR MBR		N18 I12 O10.2
Rheumatoid arthritis	LMP-365 to LMP	Categorical: Y/N.	NPR MBR		M05 M06 M08
Thyroid disease	LMP-365 to LMP	Categorical: Y/N.	NPR MBR		E00-E07

© 2024 Razaz N et al. *JAMA Neurol.*

Cohort Characteristic	Time Frame	Functional Form of Variable and Comments	Registers Used	Codes ATC	Codes ICD-10
Heart disease	LMP-365 to LMP	Categorical: Y/N.	NPR MBR		I20-I25 I30-I52 I11 O10.1
Pre-existing diabetes	LMP-365 to LMP	Categorical: Y/N.	NPR MBR		E10-E14 O24.0-O24.3
Number of chronic somatic diseases	LMP-365 to LMP	Categorical. Number of above-mentioned disease groups: 0, 1, or ≥2	NPR MBR		As listed above
**Psychiatric comorbidities**					
Depression	LMP-365 to LMP	Categorical: Y/N.<br>Includes depression and other mood disorders	NPR		F32-F39
Anxiety disorders	LMP-365 to LMP	Categorical: Y/N.	NPR		F40-F48
Personality disorders	LMP-365 to LMP	Categorical: Y/N.	NPR		F60-F69
Bipolar disorder	LMP-365 to LMP	Categorical: Y/N.<br>At least one code of bipolar disorder from either NPR and/or PDR and/or MBR, or lithium use. Codes available from PDR only in DK, FI and NO.	NPR PDR	N05AN01	F31
Schizophrenia, schizotypal and delusional disorders	LMP-365 to LMP	Categorical: Y/N.	NPR PDR		F20-F29
Any psychiatric morbidity	LMP-365 to LMP	Categorical: Y/N<br>Any of the above listed disorders or at least one prescription with antipsychotics or antidepressants		N05A, N06A	F20-F29, F32-F39, F40-F48, F60-F69

LMP: last menstrual period. MBR: Medical birth register, NPR: National patient register, PDR: Prescribed drug register, NSI: National statistical institutes, ICD-10: International statistical classification of diseases and related health problems, revision10. ATC: anatomical therapeutic chemical classification system. FI: Finland, DK: Denmark, IS: Iceland, NO: Norway, SE: Sweden. DK: ICD-10 in NPR from 1994, MBR from 1997. FI: ICD-10 in NPR (FHDR) and MBR from 1996. IS: ICD-10 from 1997. NO: ICD-10 in MBR from 1998/1999, in NPR from 2008. SE: ICD-10 in NPR and MBR from 1997.

© 2024 Razaz N et al. *JAMA Neurol.*

eTable 3: Information available in SCAN-AED from each country to determine maternal epilepsy status

	The National Patient Register	The National Prescription Register	The Medical Birth Register
Denmark	Inpatient contacts since 1994; outpatient and emergency room contacts since 1995	Information on indication for prescriptions are available since 2004. After 2004, this information is missing in app	Not used
Finland	Inpatient contacts since 1996; outpatient contacts in public hospitals since 1998	Information on indication is available when an individual is entitled to special reimbursement. Women with epilepsy are entitled to special reimbursement with specific reimbursement codes (111, 181, 182, 183, 199)	Up to 20 ICD-10 diagnostic codes available since 2004
Iceland	Inpatient contact since 2002 and outpatient contact since 2010 contacts	Not used	Up to 15 ICD-10 diagnostic codes available for each birth
Norway	Outpatient and inpatient contact and data from contracted private specialists since 2008	Information on indications for reimbursed medications since 2004	Checkbox for epilepsy diagnosis for the entire study period
Sweden	Outpatient and inpatient data since 2005	Not used	Up to 12 ICD-10 diagnostic codes available for each birth

Defining maternal epilepsy:
Defining maternal epilepsy Several data sources were used to identify maternal diagnoses of epilepsy and psychiatric disorders, including the national patient/hospital-, birth- and prescription registers (Laugesen et al. (2021), Clin Epidemiol, Vol 13 p. 533-554). Different approaches were used in each country

© 2024 Razaz N et al. *JAMA Neurol.*

depending on data availability and quality (eTable 3). Maternal epilepsy was defined by hospital contacts with epilepsy (ICD-10 G40-G41), use of ASM with epilepsy as indication or reason for reimbursement before time of birth, or diagnosis of epilepsy in the Medical Birth Register

© 2024 Razaz N et al. *JAMA Neurol.*

eTable 4. Severe maternal and neonatal morbidity, ICD-10 codes.*

Severe maternal morbidity	Severe maternal morbidity subtype	ICD 10
**Severe preeclampsia, HELLP, eclampsia**	Severe pre-eclampsia, HELP syndrome	O14.1, O14.2
	Eclampsia	O15
**Severe hemorrhage**	Antepartum hemorrhage with coagulation defect	O46.0
	Intrapartum haemorrhage with coagulation defect	O67.0
	Intrapartum haemorrhage	O67.8
	Postpartum haemorrhage	O72.3
**Surgical complications**	Cardiac complications of obstetric surgery and procedures	O75.4
	Caesarean hysterectomy	O82.2
	Disruption or hematoma of obstetric wound	O90.0, O90.1
**Sepsis**	Puerperal sepsis	O85/O85.9 (Only from MBR in Sweden)
	Septicaemia in labor	O75.3
	Streptococcal sepsis	A40
	Other sepsis	A41
**Pulmonary and obstetric embolism, DIC, shock embolism, DIC, shock**	Pulmonary and Obstetric embolism	O88, I26
	Disseminated intravascular coagulation /additionally codes to capture it	D65, O72.3
	Shock during or following labor and delivery	O75.1
**Cardiac complications**	Cardiomyopathy	O90.3, I42, I43
	Cardiac arrest and resuscitation	I46, I49.0
	Myocardial infarction	I21, I22
	Heart failure	I50
	Pulmonary edema	J81
**Acute renal failure**	Postpartum acute renal failure	O90.4
	Postprocedural renal failure	N99.0
	Acute renal failure	N17
	Unspecified kidney failure	N19

© 2024 Razaz N et al. *JAMA Neurol.*

**Cerebrovascular accidents**	Cerebral venous thrombosis in pregnancy, puerperium	O22.5, O87.3
	Subarachnoid and intracerebral hemorrhage	I60, I61
	Other non-traumatic intracranial hemorrhage	I62
	Cerebral infarction, stroke	I63, I64
**Complications of anaesthesia during pregnancy, labor and delivery, or puerperium**	Pulmonary, cardiac, CNS complications of anaesthesia during pregnancy	O29.0, O29.1, O29.2
	Pulmonary, cardiac, CNS complications of anaesthesia during labor and delivery	O74.0, O74.1, O74.2, O74.3
	Pulmonary, cardiac, CNS complications of anaesthesia during the puerperium	O89.0, O89.1, O89.2
**Severe mental health condition****	Any psychiatric or neurodevelopmental inpatient care	F00-F99
	Suicide attempt	X60-X84, Y10-Y34
	Acute psychosis	F53.1, F23

**Neonatal morbidity**	**Subtype**	**ICD 10**
Stillbirth (intrapartum or antepartum fetal death after 22 weeks)		
Neonatal death (death within 0-27 days)		
Perinatal mortality	Stillbirth or neonatal death	
**Severe neonatal morbidity**		
Intracranial hemorrhage (nontraumatic) of newborn		P52
Periventricular leukomalacia		P91.2
Hypoxic ischemic encephalopathy		P91.3-P91.6
Neonatal convulsions		P90
Retinopathy of prematurity		H35.1
RDS		P22.0
Bronchopulmonary dysplasia		P27.1
Pneumothorax		P25.1
Necrotizing enterocolitis		P77
Hypoxic ischemic encephalopathy		P96.1
Perinatal intestinal perforation		P78.0
Sepsis		P36

© 2024 Razaz N et al. *JAMA Neurol.*

Severe birth trauma	Intracranial hemorrhage/laceration	P10
	Cerebral edema	P11.0
	Brain damage	P11.1, P11.2
	Birth injury to cranial nerves (other than facial nerve)	P11.4
	Birth injury to spine and spinal cord	P11.5
	Epicranial subaponeurotic hemorrhage due to birth injury	P12.2
	Fracture of skull due to birth injury	P13.0
	Birth injury to femur, other long bones	P13.2, P13.3
	Erb's paralysis due to birth injury	P14.0
	Klumpke's paralysis due to birth injury	P14.1
	Other brachial plexus birth injuries	P14.3
	Birth injury to liver, spleen	P15.0, P15.1

*Definition includes only inpatient diagnoses from NPR and diagnostic codes registered in MBR from MLP up to 42 days after birth.

** For mental health conditions the definition included only new hospitalizations after birth with F00-F9 reported as primary diagnosis (not hospitalizations during pregnancy or diagnostic codes reported at time of birth) to distinguish from pre-existing psychiatrist conditions.. For suicide attempts and acute psychosis we used LMP up to 42 days post partum.

© 2024 Razaz N et al. *JAMA Neurol.*

eTable 5. Severe neonatal Morbidity (per 1000 Births) by Maternal Epilepsy Status and drug exposure in Five Nordic Countries (1997-2017)

	Without Epilepsy (n = 4462009)		With Epilepsy (n = 35145)			No ASMs (n = 18974)		Any ASMs (n = 16171)		
**Severe neonatal morbidity**	**No. of outcome**	**Rate per 1000 deliveries/births**	**No. of outcome**	**Rate per 1000 deliveries/ births**	**Adjusted Odds Ratios (95% CI)**	**No. of outcome**	**Rate per 1000 deliveries/births**	**No. of outcome**	**Rate per 1000 deliveries/ births**	**Adjusted Odds Ratios (95% CI)**
Intracranial hemorrhage	6705	1.5	78	2.22	1.24 (0.99-1.56)	39	2.06	39	2.41	1.12 (0.71-1.77)
Periventricular leukomalacia	354	0.08	<5	NA	NA	<5	NA	<5	NA	NA
Neonatal convulsions	7476	1.68	114	3.24	1.68 (1.39-2.03)	53	2.79	61	3.77	1.30 (0.89-1.91)
Retinopathy of prematurity	2157	0.48	33	0.94	1.44 (1.02-2.03)	20	1.05	13	0.8	0.92 (0.44-1.92)
Respiratory distress syndrome	30859	6.92	451	12.83	1.47 (1.34-1.62)	231	12.1	220	13.6	1.29 (1.05-1.57)
Bronchopulmonary dysplasia	5006	1.12	66	1.88	1.36 (1.07-1.74)	38	2	28	1.73	0.85 (0.50-1.44)
Pneumothorax	9541	2.14	109	3.1	1.41 (1.16-1.71)	55	2.9	54	3.34	0.99 (0.65-1.49)
Necrotizing enterocolitis	1621	0.36	17	0.48	1.07 (0.66-1.73)	6	0.32	11	0.68	2.24 (0.81-6.23)
Hypoxic ischemic encephalopathy	2426	0.54	233	6.63	4.39 (3.76-5.14)	103	5.43	130	8.04	1.75 (1.33-2.31)
Perinatal intestinal perforation	435	0.1	<5	NA	NA	<5	NA	<5	NA	NA
Sepsis	64308	14.4	660	18.78	1.20 (1.11-1.29)	288	15.2	372	23	1.32 (1.11-1.56)
Severe birth trauma	9667	2.17	89	2.53	1.20 (0.97-1.48)	32	1.69	57	3.52	1.65 (1.04-2.64)

[a]Data from Iceland excluded from analyses where mortality is included in the outcome because of lack of data on maternal mortality, denominator n = 4413440

[b]Stillborn children excluded from analyses of neonatal death and morbidity endpoints

ORs are exponentiated coefficients from GEE-model with binomial distribution, logit-link and robust standard errors

[d]Adjusted for maternal age, parity, birth year, child's sex, mother's education, marital status, country, and maternal psychiatric morbidity

© 2024 Razaz N et al. *JAMA Neurol.*

eTable 6. Composite severe maternal and neonatal mortality and morbidity by maternal Epilepsy Status and drug exposure stratified by maternal psychiatric comorbidity in Five Nordic Countries (1997-2017)

Outcome	Without psychiatric comorbidity (n=4263570)					With psychiatric comorbidity (n=247697)					P-value for interaction
	Without Epilepsy (n= 4233568)		With Epilepsy (n=30,002)		Adjusted OR (95% CI)*	Without Epilepsy (n=242416)		With Epilepsy (n=5281)		Adjusted OR (95% CI)*	
	No.	Rate per 1000	No.	Rate per 1000		No.	Rate per 1000	No.	Rate per 1000		
Composite Maternal death/maternal morbidity	102052	24.4	1004	33.6	1.25 (1.17-1.33)	10063	42.8	290	55.5	1.21 (1.07-1.37)	0.39
**Fetal/infant mortality and morbidity**											
Composite perinatal death/severe neonatal morbidity	123476	29.2	1300	43.3	1.41 (1.33-1.49)	9514	39.3	372	70.4	1.57 (1.41-1.76)	0.062

*Adjusted for maternal age, parity, birth year, child's sex mother's education, marital status, country, number of chronic conditions and number of prepregnancy hospitalizations

© 2024 Razaz N et al. *JAMA Neurol.*

eTable 7. Maternal and Perinatal Mortality and Severe Morbidity by Maternal Epilepsy Status in Five Nordic Countries adjusting for adjusting for, maternal BMI and smoking in early pregnancy (1997-2017)

Outcome	Model 1	Model 2
Maternal mortality[a]	3.86 (1.84-8.10)	3.81 (1.81-8.00)
Composite severe maternal morbidity	1.23 (1.16-1.31)	1.22 (1.15-1.29)
Severe preeclampsia, HELLP, Eclampsia	1.30 (1.19-1.42)	1.28 (1.18-1.40)
Severe Hemorrhage	1.05 (0.93-1.19)	1.05 (0.92-1.19)
Pulmonary and obstetric embolism, DIC, shock	1.42 (1.05-1.93)	1.40 (1.03-1.91)
Sepsis	1.03 (0.90-1.17)	1.02 (0.89-1.16)
Acute renal failure	1.15 (0.54-2.42)	1.15 (0.54-2.43)
Cardiac complications	1.16 (0.70-1.90)	1.15 (0.70-1.89)
Complications of anesthesia	NA	NA
Cerebrovascular accidents	5.81 (4.27-7.89)	5.71 (4.20-7.75)
Surgical complications	1.23 (0.90-1.66)	1.21 (0.89-1.64)
Severe mental health conditions	1.81 (1.50-2.19)	1.79 (1.48-2.16)
Uterine rupture	1.02 (0.74-1.39)	1.01 (0.74-1.38)
Composite Maternal death/maternal morbidity	1.23 (1.16-1.31)	1.22 (1.15-1.29)
**Fetal/infant mortality and morbidity[b]**		
Stillbirth	1.18 (1.00-1.40)	1.16 (0.98-1.38)
Neonatal death	1.23 (0.98-1.55)	1.21 (0.96-1.52)
Perinatal death	1.20 (1.05-1.38)	1.18 (1.03-1.35)
Composite severe neonatal morbidity[f]	1.48 (1.40-1.56)	1.46 (1.38-1.54)
Composite perinatal death/severe neonatal morbidity[f]	1.44 (1.37-1.52)	1.42 (1.35-1.49)

Abbreviation: NA, not applicable, DIC denotes disseminated intravascular coagulation.
[a]Data from Iceland excluded from analyses where mortality is included in the outcome because of lack of data on maternal mortality,
[b]Stillborn children excluded from analyses of neonatal death and morbidity endpoints
[c]ORs are exponentiated coefficients from GEE-model with binomial distribution, logit-link and robust standard errors
[d]Adjusted for maternal age, parity, birth year, child's sex mother's education, marital status, country, maternal psychiatric morbidity
[e]NA, owing to personal data protection restriction on publishing cell counts less than 5.
[f] Includes respiratory distress syndrome, retinopathy of prematurity, intraventricular hemorrhage (grade 3 or more), intracranial hemorrhage, sepsis, necrotizing enterocolitis, severe birth trauma, and seizures
Model 1: Adjusted for maternal age, parity, birth year, child's sex, mother's education, marital status, country, pre-pregnancy maternal psychiatric morbidity, number of chronic conditions and number of prepregnancy hospitalizations
Model 2: Adjusted for maternal age, parity, birth year, child's sex, mother's education, marital status, country, pre-pregnancy maternal psychiatric morbidity, number of chronic conditions, number of prepregnancy hospitalizations, pre-pregnancy BMI and smoking in early pregnancy

© 2024 Razaz N et al. *JAMA Neurol.*

Table e8. Maternal and Perinatal Mortality and Severe Morbidity Among Women with Epilepsy by Antiseizure Medications (ASMs) Use During Pregnancies adjusting for maternal BMI and smoking in early pregnancy (1997-2017)

Outcome	Model 1	Model 2
Maternal mortality[a]	NA	NA
Composite severe maternal morbidity	1.24 (1.10-1.40)	1.23 (1.09-1.39)
Severe preeclampsia, HELLP, Eclampsia	1.23 (1.03-1.48)	1.23 (1.02-1.48)
Severe Hemorrhage	1.61 (1.24-2.10)	1.60 (1.23-2.09)
Pulmonary and obstetric embolism, DIC, shock	1.55 (0.84-2.87)	1.56 (0.84-2.89)
Sepsis	0.90 (0.68-1.20)	0.90 (0.68-1.19)
Acute renal failure	NA	NA
Cardiac complications	0.74 (0.23-2.39)	0.73 (0.22-2.39)
Complications of anesthesia	NA	NA
Cerebrovascular accidents	1.99 (1.13-3.50)	1.98 (1.12-3.50)
Surgical complications	1.14 (0.64-2.03)	1.14 (0.64-2.03)
Severe mental health conditions	1.28 (0.87-1.89)	1.26 (0.86-1.86)
Uterine rupture	1.07 (0.56-2.04)	1.08 (0.57-2.05)
Composite Maternal death/maternal morbidity	1.24 (1.10-1.40)	1.23 (1.09-1.39)
**Fetal/infant mortality and morbidity[b]**		
Stillbirth	1.05 (0.72-1.54)	1.05 (0.72-1.54)
Neonatal death	2.40 (1.44-4.00)	2.38 (1.43-3.97)
Perinatal death	1.40 (1.03-1.89)	1.39 (1.03-1.88)
Composite severe neonatal morbidity[f]	1.37 (1.22-1.53)	1.36 (1.22-1.53)
Composite perinatal death/severe neonatal morbidity[f]	1.37 (1.23-1.52)	1.36 (1.22-1.52)

Abbreviation: NA, not applicable, DIC denotes disseminated intravascular coagulation.
[a]Data from Iceland excluded from analyses where mortality is included in the outcome because of lack of data on maternal mortality,
[b]Stillborn children excluded from analyses of neonatal death and morbidity endpoints
[c]ORs are exponentiated coefficients from GEE-model with binomial distribution, logit-link and robust standard errors
[d]Adjusted for maternal age, parity, birth year, child's sex mother's education, marital status, country, maternal psychiatric morbidity
[e]NA, owing to personal data protection restriction on publishing cell counts less than 5.
[f] Includes respiratory distress syndrome, retinopathy of prematurity, intraventricular hemorrhage (grade 3 or more), intracranial hemorrhage, sepsis, necrotizing enterocolitis, severe birth trauma, and seizures
Model 1: adjusted for maternal age, parity, birth year, child's sex, mother's education, marital status, country, pre-pregnancy maternal psychiatric morbidity, number of chronic conditions and number of prepregnancy hospitalizations
Model 2: adjusted for maternal age, parity, birth year, child's sex, mother's education, marital status, country, pre-pregnancy maternal psychiatric morbidity, number of chronic conditions, number of prepregnancy hospitalizations, pre-pregnancy BMI and smoking in early pregnancy

© 2024 Razaz N et al. *JAMA Neurol.*

Table e9: Maternal and perinatal severe morbidity by maternal epilepsy status and ASM exposure, stratified by country

	Without Epilepsy		With epilepsy			No ASM		Any ASM		
Outcome	**No. of event**	**Rate per 1000**	**No. of event**	**Rate per 1000**	**Adjusted OR (95% CI)***	**No. of event**	**Rate per 1000**	**No. of event**	**Rate per 1000**	**Adjusted OR (95% CI)***
**Composite severe maternal morbidity**										
Denmark	23394	18.97	336	29.55	1.32 (1.18-1.47)	194	28.86	142	30.53	1.13 (0.90-1.41)
Finland	23225	19.83	184	31.50	1.42 (1.21-1.65)	29	24.85	155	33.16	1.24 (0.82-1.89)
Iceland	1339	22.77	10	38.31	1.55 (0.82-2.96)	<5	NA	<10	39.11	1.39 (0.29-6.74)
Norway	22786	30.89	356	41.44	1.19 (1.06-1.33)	230	40.01	126	44.33	1.14 (0.91-1.44)
Sweden	42611	33.43	412	44.69	1.18 (1.06-1.31)	200	37.56	212	54.44	1.48 (1.20-1.83)
**Composite severe neonatal morbidity****										
Denmark	30555	24.85	486	42.89	1.50 (1.36-1.65)	238	35.54	248	53.52	1.55 (1.28-1.87)
Finland	37911	32.46	321	55.28	1.54 (1.36-1.73)	49	42.35	272	58.49	1.46 (1.05-2.04)
Iceland	1539	26.22	12	46.51	1.59 (0.91-2.79)	<5	NA	<12	62.50	NA
Norway	20832	28.33	405	47.29	1.48 (1.34-1.65)	250	43.61	155	54.75	1.26 (1.02-1.56)
Sweden	23828	18.75	272	29.61	1.41 (1.24-1.59)	146	27.52	126	32.47	1.13 (0.88-1.46)
**Composite perinatal death/severe neonatal morbidity**										
Denmark	35867	29.08	539	47.40	1.47 (1.35-1.61)	266	39.58	273	58.70	1.53 (1.28-1.84)
Finland	42779	36.52	370	63.35	1.59 (1.42-1.78)	60	51.41	310	66.32	1.37 (1.00-1.86)
Iceland	1693	28.79	15	57.47	1.79 (1.09-2.94)	<5	NA	<15	78.21	NA
Norway	23796	32.25	434	50.52	1.40 (1.26-1.55)	265	46.10	169	59.47	1.30 (1.05-1.59)
Sweden	28855	22.64	314	34.06	1.35 (1.20-1.52)	168	31.55	146	37.49	1.14 (0.90-1.44)

*Adjusted for maternal age, parity, birth year, child's sex, mother's education, marital status, country, pre-pregnancy maternal psychiatric morbidity, number of chronic conditions and number of prepregnancy hospitalizations
**Stillborn children excluded from analyses of neonatal death and morbidity endpoints

© 2024 Razaz N et al. *JAMA Neurol.*

Table e10: Maternal severe preeclampsia or HELLP (excluding eclampsia)

	Without Epilepsy		With epilepsy			No ASM		Any ASM		
Outcome	**No. of event**	**Rate per 1000**	**No. of event**	**Rate per 1000**	**Adjusted OR (95% CI)***	**No. of event**	**Rate per 1000**	**No. of event**	**Rate per 1000**	**Adjusted OR (95% CI)***
**Severe preeclampsia, HELLP**	45795	10.23	527	14.94	1.27 (1.16-1.38)	263	13.81	264	16.26	1.27 (1.06-1.53)

*Adjusted for maternal age, parity, birth year, child's sex, mother's education, marital status, country, maternal psychiatric morbidity, number of chronic conditions and number of prepregnancy hospitalizations

© 2024 Razaz N et al. *JAMA Neurol.*

© 2024 Razaz N et al. *JAMA Neurol.*

Table e11: Composite severe maternal and neonatal mortality and morbidity by maternal Epilepsy Status, restricting the epilepsy diagnosis within 1 year before the date of conception

Outcomes	Without Epilepsy (n = 4486438)		With Epilepsy (n = 4829)		
	**No. of events**	**Rate per 1000 deliveries/births (95% CI)**	**No. of events**	**Rate per 1000 deliveries/births (95% CI)**	**Adjusted Odds Ratios (95%CI)[c]**
Composite maternal mortality/maternal morbidity[a]	112473	25.40(25.26-25.55)	933	37.94(35.62-40.40)	1.26 (1.17-1.35)
Maternal mortality[a]	206	0.05(0.04-0.05)	8	0.33(0.16-0.65)	5.43 (2.61-11.29)
Composite severe maternal morbidity	113713	25.35(25.20-25.49)	940	37.86(35.56-40.31)	1.26 (1.18-1.35)
Severe preeclampsia, HELLP, Eclampsia	47485	10.58(10.49-10.68)	419	16.88(15.35-18.55)	1.37 (1.24-1.51)
Severe Hemorrhage	28588	6.37(6.30-6.45)	208	8.38(7.32-9.59)	1.06 (0.92-1.22)
Pulmonary and obstetric embolism, DIC, shock	3270	0.73(0.70-0.75)	32	1.29(0.91-1.82)	1.46 (1.03-2.07)
Sepsis	22807	5.08(5.02-5.15)	136	5.48(4.63-6.48)	0.93 (0.79-1.10)
Acute renal failure	483*	0.11(0.10-0.12)	7	0.20(0.08-0.48)	1.19 (0.49-2.89)
Cardiac complications	1206	0.27(0.25-0.28)	12	0.48(0.27-0.85)	1.15 (0.63-2.07)
Complications of anesthesia	429	0.10(0.09-0.11)	<5	NA	NA
Cerebrovascular accidents	974	0.22(0.20-0.23)	53*	1.97(1.49-2.61)	7.73 (5.69-10.50)
Surgical complications	3698	0.82(0.80-0.85)	27	1.09(0.75-1.59)	1.17 (0.80-1.72)
Severe mental health conditions	4616	1.03(1.00-1.06)	77	3.10(2.48-3.88)	1.76 (1.39-2.22)
Uterine rupture		1.10(1.07-1.13)	26	1.05(0.71-1.54)	0.96 (0.65-1.41)
**Fetal/infant mortality and morbidity[b]**					
Composite perinatal mortality/severe neonatal morbidity[e]	133470	29.75(29.59-29.91)	1192	48.01(45.42-50.74)	1.48 (1.39-1.57)
Stillbirth[f] (after 22 weeks gestation)	14010	3.12(3.07-3.17)	103	4.15(3.42-5.03)	1.26 (1.03-1.53)
Neonatal death [g] (0-27 days)	7051	1.58(1.54-1.61)	59	2.39(1.85-3.08)	1.41 (1.09-1.82)
Perinatal mortality[f] *(stillbirth and neonatal death)	21061	4.69(4.63-4.76)	162	6.52(5.60-7.61)	1.31 (1.12-1.53)
Composite severe neonatal morbidity[e,g]	115107	25.74(25.59-25.88)	1054	42.63(40.18-45.22)	1.50 (1.41-1.60)

Abbreviation: NA, not applicable, DIC denotes disseminated intravascular coagulation.

[a]Data from Iceland excluded from analyses where mortality is included in the outcome because of lack of data on maternal mortality

[b]Stillborn children excluded from analyses of neonatal death and morbidity endpoints

[c]ORs are exponentiated coefficients from GEE-model with binomial distribution, logit-link and robust standard errors. Adjusted for maternal age, parity, birth year, child's sex, mother's education, marital status, country, maternal psychiatric morbidity, number of chronic conditions and number of prepregnancy hospitalizations

[d]NA, owing to personal data protection restriction on publishing cell counts less than 5

e Includes respiratory distress syndrome, retinopathy of prematurity, intraventricular hemorrhage (≥grade 3), intracranial hemorrhage, sepsis, necrotizing enterocolitis, severe birth trauma, and seizures

[f] Stillbirths, perinatal deaths and composite perinatal death/severe neonatal morbidity are expressed per 1000 total births

[g] Neonatal deaths and composite severe neonatal morbidity are expressed per 1000 live births

© 2024 Razaz N et al. *JAMA Neurol.*

© 2024 Razaz N et al. *JAMA Neurol.*
