# Supplementary material for: Risk of Perinatal and Maternal Morbidity and Mortality Among Pregnant Women With Epilepsy
Source: JAMA Neurol. 2024 Aug 5;81(9):985–95. doi: 10.1001/jamaneurol.2024.2375 (PMC11385047; doi:10.1001/jamaneurol.2024.2375)
Supplement: Supplement 2. — Data sharing statement [file jamaneurol-e242375-s002.pdf]

Data Sharing Statement

Razaz. Risk of Perinatal and Maternal Morbidity and Mortality Among Pregnant Women With Epilepsy. *JAMA Neurol*. Published August 05, 2024. doi:10.1001/jamaneurol.2024.2375

Data
Data available: No
